# The capsaicin binding affinity of wildtype and mutant TRPV1 ion channels

**DOI:** 10.1016/j.jbc.2023.105268

**Published:** 2023-09-20

**Authors:** Shisheng Li, Jie Zheng

**Affiliations:** Department of Physiology and Membrane Biology, University of California at Davis, School of Medicine, Davis California, USA

**Keywords:** nociception, allostery, cooperativity, concatemer, resiniferatoxin

## Abstract

Vanilloids such as capsaicin and resiniferatoxin are highly selective and potent activators for transient receptor potential vanilloid subfamily, member 1, a nociceptor for heat and pain perception. However, the intrinsic vanilloid binding affinity, key for understanding transient receptor potential vanilloid subfamily, member 1 function, remains unknown despite intensive investigations by electrophysiological, structural, and computational methods. In this study, we determined capsaicin binding affinity under physiological conditions by isolating individual binding steps to each subunit with concatemers. We estimated the capsaicin association constant of a wildtype subunit to be in the order of 10^6^ M^−1^ and that of the Y511A mutant subunit to be a hundred times lower, in the order of 10^4^ M^−1^. The Y511A mutation, located at the entrance of the vanilloid binding pocket, reduces binding affinity without a noticeable effect on activation gating. We further affirmed that there is little cooperativity between vanilloid binding steps. Models based on independent binding and equally cooperative subunit gating can accurately describe capsaicin activation.

Capsaicin receptor transient receptor potential vanilloid subfamily, member 1 (TRPV1) ion channel is a pivotal sensor of harmful heat and protons as well as the piquant sensation linked to spicy foods (1, 2). Its activation by vanilloids such as capsaicin and resiniferatoxin (RTX) underlines an important form of nociception and offers a pathway for developing new analgesics (3). Vanilloids bind to the vanilloid binding pocket located within the transmembrane domain of the channel, formed by S3, S4, and the S4–S5 linker from one subunit and S5 and S6 from a neighbor subunit (4, 5) (Fig. 1*A*). As a homotetramer, each TRPV1 channel can accommodate four vanilloid molecules that cooperatively activate the channel (1, 4, 5). Vanilloid-induced TRPV1 activation has been serving as an outstanding model system for elucidating the energetics and kinetics of ligand activation, thanks to extensive structural (4, 5, 6, 7, 8, 9), functional (10, 11, 12, 13, 14, 15, 16, 17, 18, 19), and computational investigations (20, 21). Nonetheless, a fundamental feature of TRPV1 activation—the binding affinity of capsaicin and other vanilloids—has not been determined. The EC_50_ value, a descriptive term combining binding and activation (22), has been generally used.

**Figure 1:**
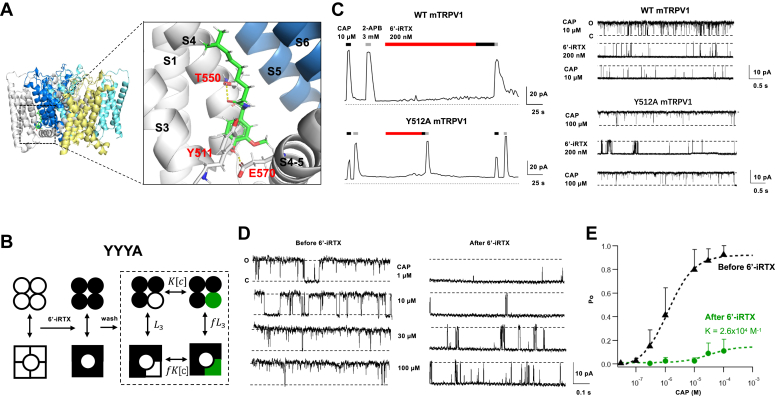
**Direct measurement of capsaicin binding affinity to the Y511A mutant binding site.***A*, structure of the rat TRPV1 (based on PDB entry: 3J5R), with a zoom-in view of the vanilloid binding pocket, capsaicin (*green*), and key interacting residues. *Yellow dashed lines* indicate a hydrogen bond. *B*, illustration of the experimental scheme for the YYYA concatemer. A solution of 200 nm 6′-iRTX was first perfused to a membrane patch containing a single YYYA channel to occupy all the binding sites; after washing off the reversibly bound 6′-iRTX from the A subunits, capsaicin solutions at varying concentrations were perfused to occupy the available binding site in the mutant A subunit. The *dashed box* highlights an allosteric model for capsaicin binding to the single binding site. L_3_, K[c], fL_3_, and fK[c] are equilibrium constants for the respective transition. *C*, representative macroscopic (*left panel*) and single-channel (*right panel*) inside–out patch-clamp recordings from wildtype and Y512A mutant mouse TRPV1 (equivalents to Y511A rat TRPV1). *D*, representative capsaicin responses of a YYYA channel before (*left*) and after (*right*) loading the channel’s Y subunits with 6′-iRTX. *E*, summarized capsaicin-dependent Po with or without 6′-iRTX in the Y subunits, data are reported as mean ± SD. *Black triangles*: capsaicin responses without 6′-iRTX, fitted to a Hill equation with the following parameters: EC_50_ = 1.2 × 10^−6^ M, Hill slope = 0.96. N = 5 to 9. *Green circles*: capsaicin responses with preloaded 6′-iRTX in the Y subunits, fitted to the allosteric model derived for the allosteric system shown in panel *B* with the following parameters: L_3_ = 0.1, f = 15.4, K = 2.6 × 10^4^ M^−1^. N = 5. 6′-iRTX, 6′-iodo-RTX; PDB, Protein Data Bank; Po, open probability; TRPV1, transient receptor potential vanilloid subfamily, member 1.

Activation of TRPV1 by vanilloids is an allosteric process for which the classic Monod–Wyman–Changeux (MWC) model can be applied (23, 24) (Fig. S1). The MWC model is defined by three key parameters: the apo-state equilibrium constant (L_0_), activation energy from each ligand binding represented by the cooperative factor (f), and the ligand association constant (K). A quantitative understanding of the vanilloid activation process requires knowledge of these three parameters. L_0_, reflecting the resting activity level, has been measured by many groups; our estimates from mouse TRPV1 channels range from 0.005 to 0.007 (12, 24). In a recent study, we confirmed that the activation energies for the four vanilloid-binding steps are virtually the same and determined the value to be 1.70 to 1.86 kcal/mol for RTX (24). The present study aimed to determine the value for ligand binding affinity.

Capsaicin is an extremely pungent compound. It activates TRPV1 in submicromolar concentrations under physiological conditions (3). To define the capsaicin binding process entails answering three essential and closely interconnected questions: (1) What is the binding affinity of capsaicin for each subunit? (2) Does binding of capsaicin to the four subunits occur independently or cooperatively? (3) How do residues in the vanilloid binding pocket contribute to capsaicin binding? Addressing these questions is a challenging task. With four (wildtype or mutant) binding sites per channel and the possibility of cooperative binding and activation, a general binding model would contain up to 32 states, each with a distinct binding affinity. To overcome this challenge, our approach was to first preload some of the binding sites in an irreversible manner, allowing us to measure capsaicin binding to one or two remaining binding sites.

Preventing capsaicin binding in individual subunits can be potentially achieved by introducing mutations to the binding site. However, no such mutation has been found. Moreover, the approach would completely obliterate the binding site and, more importantly, have a potential global impact to activation. To circumvent these confounding issues, we took advantage of the irreversible binding of RTX and its derivatives to block out selected subunits (whose functional contribution to activation could still be quantified). Previous studies have demonstrated that the rat TRPV1 Y511A mutation (equivalent to Y521A in mouse TRPV1) could convert RTX binding from irreversible to reversible without significantly altering activation gating (12, 16, 24, 25). In an earlier study, it has been shown that different combinations of wildtype and Y511A subunits in concatemers would allow faithful introduction of these phenotypes in a stoichiometric manner (25). Building upon these findings, we developed a method to block two or three binding sites in order to quantify capsaicin binding affinity to the remaining binding site(s) with single-channel measurements of channel open probability (Po). Once capsaicin binding affinity to the Y511A subunit was known, we could then determine the capsaicin binding affinity to a wildtype subunit. The possibility of cooperative bindings was examined and ruled out in the process.

## Results

### Direct functional measurement of capsaicin binding affinity to a single subunit

In a previous study, we found that RTX binds irreversibly to wildtype (Y) TRPV1 subunits but can be washed off Y511A (A) mutant subunits (24). Therefore, if we first treat YYYA concatemeric channels with RTX and then wash thoroughly, RTX will occupy the vanilloid binding sites of the three wildtype subunits. We can then directly measure association constant (K) and energy contribution (f) of capsaicin from the single mutant binding site (Fig. 1*B*). However, when three RTX molecules were bound in a YYYA concatemeric channel, its Po already approached approximately 99% (24). There was hardly any observable change for the subsequent capsaicin binding step. Hence, we searched for an alternative to RTX that retains the irreversible binding property but functions as a weaker TRPV1 activator. This search led us to the RTX derivative 6′-iodo-RTX (hereafter called 6′-iRTX), with an iodine atom at the 6′ position of the vanillyl group (Fig. S2), which is known as a weak partial agonist for TRPV1 (26).

In both macroscopic and single-channel recordings, we confirmed that 6′-iRTX binds irreversibly to the wildtype subunits (Figs. 1*C* and S3). This is evident from the observation that, after perfusing 6′-iRTX to wildtype mTRPV1 channels—which produced weak channel activities, as anticipated for a partial agonist—capsaicin lost its activation effect, whereas 2-aminoethoxydiphenyl borate (2-APB) could still activate these channels (Fig. 1*C*, *top panels*). In contrast, 6′-iRTX could be washed off Y512A mTRPV1 mutant channels, after which capsaicin was able to reactivate them (Fig. 1*C*, *bottom panels*; Fig. S4). With three 6′-iRTX molecules bound in a YYYA concatemer, the channel was barely activated. This allowed us to observe a subsequent increase in Po in a capsaicin concentration-dependent manner as capsaicin bound to the single remaining site (Figs. 1, *D* and *E* and S5). By fitting the concentration dependence of Po to a simple four-state model (indicated by a *dashed box* in Fig. 1*B*), we obtained the association constant for capsaicin in the Y511A mutant subunit to be 2.6 × 10^4^ M^−1^. This value is in agreement with the EC_50_ value of 1.3 × 10^−5^ M observed from the Y512A mutant mTRPV1 channels (12).

### Functional measurement of capsaicin binding affinity to two subunits

**Figure 2:**
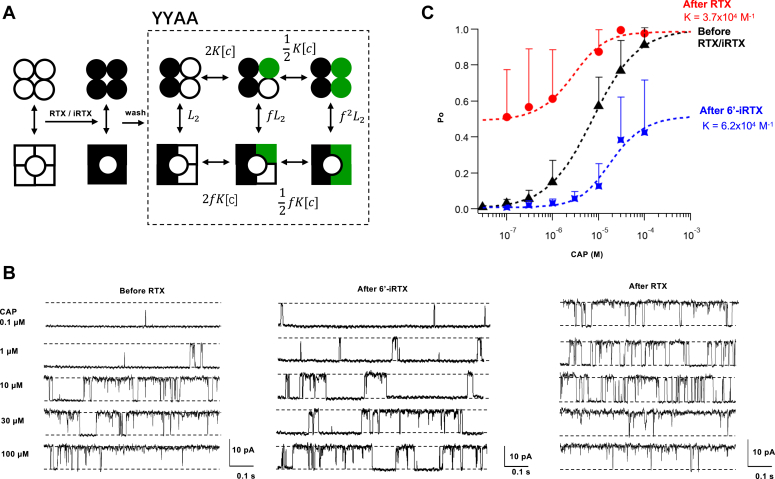
**Measurement of capsaicin binding affinity from two Y511A mutant subunits in the channel.***A*, illustration of the experimental scheme for the YYAA concatemer. A solution of 200 nm 6′-iRTX or RTX was first perfused to occupy all the binding sites; after washing off the reversibly bound 6′-iRTX or RTX from the A subunits, capsaicin solutions were perfused to occupy the available binding sites in the A subunits. The *dashed box* highlights an allosteric model for capsaicin binding to the two A subunits. The equilibrium constants for each transition are labeled. *B*, representative inside–out patch-clamp recordings from a YYAA channel before (*left*) and after loading the Y subunits with 6′-iRTX (*middle*) or RTX (*right*). *C*, summarized Po of a YYAA channel with or without RTX/6′-iRTX. Data are reported as mean ± SD. *Black triangles*: capsaicin responses without 6′-iRTX or RTX, fitted to a Hill equation with the following parameters: EC_50_ = 7.4 × 10^−6^ M, Hill slope = 0.88. N = 4 to 7. *Red circles*: capsaicin responses with preloaded RTX in the two Y subunits, fitted to the allosteric model derived from *A* with the following parameters: L_2_ = 0.96, f = 8.9, K = 3.7 × 10^4^ M^−1^. N = 5. *Blue squares*: capsaicin responses with preloaded 6′-iRTX in the Y subunits, fitted with the following parameters: L_2_ = 0.008, f = 11.8, K = 6.2 × 10^4^ M^−1^. N = 5. 6′-iRTX, 6′-iodo-RTX; Po, open probability.

The same approach described above for a single binding site can be applied to two binding sites offered by the YYAA concatemer, as illustrated in Figure 2*A*. Indeed, whereas the range of Po change we could observe from capsaicin binding to a single subunit in YYYA was relatively narrow—from about 1% to 15% (Fig. 1*E*), the range became much broader when capsaicin bound to two subunits in YYAA to increase channel activity, from about 1% to 50% (Figs. 2*B*, S6, and S7). Using the six-state model with an independent binding assumption shown in Figure 2*A*, we estimated the K value to be 6.2 × 10^4^ M^−1^ (Fig. 2*C*). This value is very close to the K value estimated for the single binding site in YYYA (Fig. 1*E*).

With only two wildtype subunits in YYAA, we had the option to preload the wildtype binding sites with the more potent agonist RTX, producing an initial Po level of approximately 50% (Fig. 2*B*). Application of capsaicin at varying concentrations further increased Po up to nearly 100%. Measurements from RTX-loaded channels served as a nice internal control as they were done at otherwise identical conditions as those with 6′-iRTX (Fig. S7). Using the same six-state model, we estimated the K value to be 3.7 × 10^4^ M^−1^ for RTX-preloaded channels. This value is in close agreement with the independently obtained values with 6′-iRTX from YYAA and YYYA. Therefore, we conclude that the Y511A mutant subunit has a capsaicin association constant of 2.6 × 10^4^ to 6.2 × 10^4^ M^−1^.

### Are capsaicin binding steps independent or cooperative?

**Figure 3:**
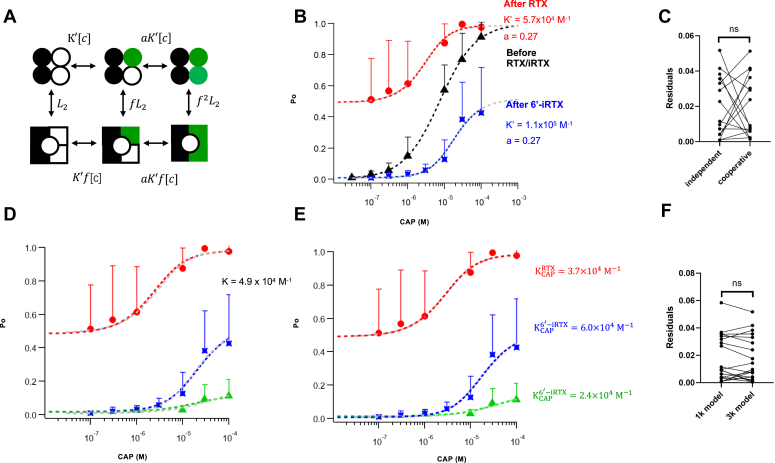
**Testing cooperativity in capsaicin bindings.***A*, an allosteric model for the YYAA concatemer, with the two capsaicin binding steps having independent association constants. *B*, global fitting of YYAA channel’s capsaicin responses using the model shown in *A*, assuming that the f and *a* values are identical when the wildtype binding sites are occupied by RTX (*red circles*) or 6′-iRTX (*blue squares*). L_2_ = 0.97 (for RTX), L_2_ = 0.01 (for 6′-iRTX), and f =10.3. *Gray curves* represent fitting results with the independent binding model in Figure 2*A*. *C*, paired Student’s *t* test for the residuals from independent binding model and cooperative binding model. *p* = 0.98. ns, no significant difference. *D*, global fitting of capsaicin concentration–dependent Po changes measured from YYYA preloaded with 6′-iRTX (*green triangles*), YYAA preloaded with RTX (*red circles*), or 6′-iRTX (*blue asterisks*). The K value for capsaicin binding was assumed to be the same for all these cases. K = 4.9 × 10^4^ M^−1^, f = 8.0, L with two prebound RTX = 0.94, L with two prebound 6′-iRTX = 0.02, and L with three prebound 6′-iRTX = 0.02. *Gray curves* represent fitting results with the independent binding model in Figures 1*B* and 2A. *E*, global fitting for capsaicin concentration–dependent Po changes assuming that the K value for capsaicin binding is different in each case. With two prebound RTX: K = 3.7 × 10^4^ M^−1^, f = 8.9, and L = 0.96; with three prebound 6′-iRTX: K = 2.4 × 10^4^ M^−1^, f = 12.1, and L = 0.01; with two prebound 6′-iRTX: K = 6.0 × 10^4^ M^−1^, f = 10.7, and L = 0.01. *Gray curves* represent fitting results with the independent binding model in Figures 1*B* and 2A. *F*, paired Student’s *t* test for the fitting residuals from the two assumptions (1K model *versus* 3K model). *p* = 0.36. 6′-iRTX, 6′-iodo-RTX; ns, no significant difference; Po, open probability.

The nearly identical K estimates from one or two binding sites strongly indicate that capsaicin bindings to different subunits of a TRPV1 channel are likely independent events. To test this hypothesis, we relaxed the independence assumption for the six-state system and assigned the capsaicin binding steps with two separate association constants, K′ and *a*K’ (Fig. 3*A*); here K′ is equivalent to 2K in Figure 2*A*. For independent bindings, the *a* value would be 0.25 (Fig. 2*A*); *a* > 0.25 or *a* < 0.25 would indicate bindings with positive or negative cooperativity, respectively. Simultaneously fitting the data from YYAA channels with preloaded RTX or 6′-iRTX using the new model yielded satisfactory concentration dependence predictions (Fig. 3*B*). The fitting residuals (differences between model and data) exhibited no detectable difference from those using the model with independent binding steps (Fig. 3*C*), even though the new model has one additional free parameter. A value of 0.27 for the *a* factor yielded the best fitting results, confirming that cooperativity between capsaicin binding steps, if existed, was too weak to be detected, and an independent model is sufficient to describe capsaicin activation.

To further check for potential binding cooperativity, we performed global fittings of the data from both YYYA and YYAA concatemers assuming the same K value for capsaicin binding, that is, binding of capsaicin was independent of what had been preloaded in the other binding sites (RTX or 6′-iRTX) and independent of the number of preloaded ligands per channel. As shown in Figure 3*D*, the approach yielded fitting results nearly indistinguishable from the individual fittings in Figures 1*E* and 2D. The K value estimated this way is also in close agreement with those determined individually. These observations suggest that binding of capsaicin is insensitive to what and how many ligands bind to the other subunits. Indeed, a global fitting with capsaicin binding being dependent on what were in the other subunits did not significantly improve the fitting outcome (Fig. 3, *E* and *F*), even though four additional free parameters were added. It is further noticed that the binding affinity estimates for the three cases differ by less than three folds from each other and from estimates using previous methods (Fig. 3*E*).

In summary, our observations suggest that there is no detectable cooperativity between bindings of vanilloid to the TRPV1 subunits.

### Binding affinity of capsaicin to a wildtype subunit

**Figure 4:**
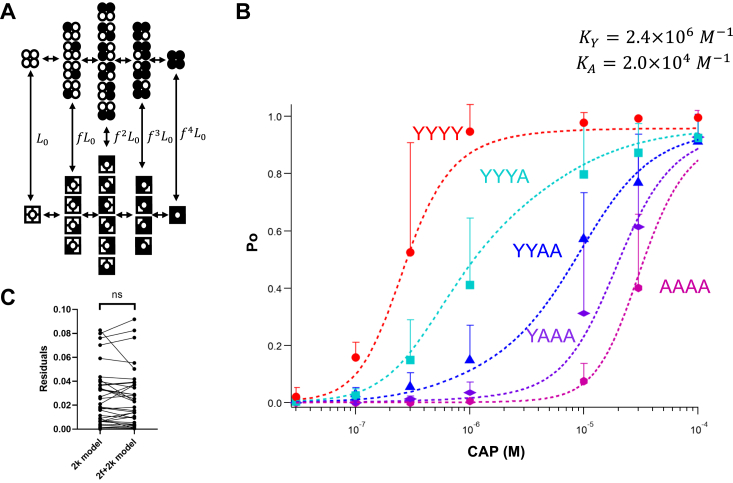
**Determining capsaicin binding affinity to the wildtype binding site.***A*, a 32-state allosteric activation model, with reversible binding for capsaicin in both mutant and wildtype subunits. *B*, global fitting of capsaicin concentration–dependent Po values from YYYY, YYYA, YYAA, YAAA, and AAAA concatemers. Data are reported as mean ± SD. Independent binding and identical f factor for Y and A subunits were assumed; the association constants for the Y and A subunits, K_Y_ and K_A_, are distinct. L_0_ = 0.001, K_Y_ = 2.4 × 10^6^ M^−1^, K_A_ = 2.0 × 10^4^ M^−1^, and f = 12.1. N = 5 to 13. *C*, paired Student’s *t* test between the residuals from fitting a 2K model and those from fitting a 2f + 2K model (in which the f factors for Y and A subunits were assumed to be different). *p* = 0.49. For fitting this 2f + 2K model, the following parameters were used: L_0_ = 0.002, K_Y_ = 2.4 × 10^6^ M^−1^, K_A_ = 1.5 × 10^4^ M^−1^, f_Y_ = 10.7, and f_A_ = 12.9. ns, no significant difference; Po, open probability.

Since models with independent ligand binding steps could accurately predict the behavior of concatemeric channels, it is possible to determine the binding affinity of capsaicin to a wildtype subunit. We approach this goal by globally fitting capsaicin activation data from concatemers with all possible combinations of wildtype and mutant subunits, without pretreatment by RTX or 6′-iRTX. For the global fitting, there were two capsaicin association constants, one for the wildtype subunit (K_Y_) and another for the mutant subunit (K_A_). The 32-state independent binding model (Fig. 4*A*) contains only four free parameters: in addition to K_Y_ and K_A_, L_0_ represents the equilibrium constant for spontaneous opening of an unliganded channel (which has been determined in our previous study (24)), and f represents the fold increase of the closed-to-open equilibrium constant because of each capsaicin binding. Fitting of this simple model simultaneously to capsaicin concentration–dependent Po curves for YYYY, YYYA, YYAA, YAAA, and AAAA concatemers yielded satisfactory results (Fig. 4*B* and S8). We found that relaxing the model to incorporate different f factors for wildtype and mutant subunits would not improve fitting outcome even with the additional free parameter (Fig. 4*C*), in agreement with the prediction that Y511 participates mainly in ligand binding but not activation gating (12, 16). The estimate for K_A_ is 2.0 × 10^4^ M^−1^, which is again in close agreement with results for the Y511A subunit discussed earlier. The estimate for capsaicin binding affinity to the wildtype subunit, K_Y_, is 2.4 × 10^6^ M^−1^. It is two orders of magnitude higher than K_A_, consistent with the observation that the Y511A mutation substantially shifted capsaicin responses to higher concentrations in a mutant number–dependent manner (25).

## Discussion

**Figure 5:**
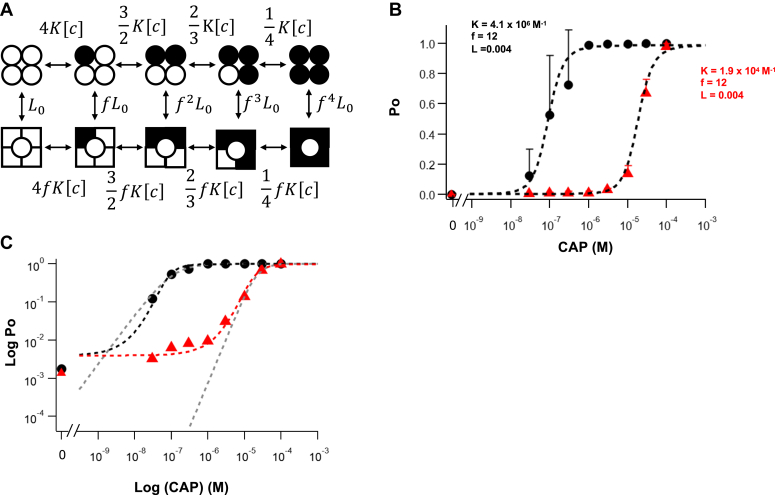
**Quantitative description of capsaicin activation of the mouse TRPV1 wildtype and mutant channels.***A*, a complete MWC model with independent ligand binding steps. *B*, concentration-dependent Po data for the wildtype and mutant channels (*black* and *red symbols*, respectively) measured at the single-channel level, Data are reported as mean ± SD, fitted to the MWC model (*dashed curves*) with the following parameters: L_0_ = 0.004, K_Y_ = 4.1 × 10^6^ M^−1^, K_A_ = 1.9 × 10^4^ M^−1^, and f = 12. *C*, log transformation of the plot shown in *B*, and overlapping Hill functions (*gray dashed curves*) with the following parameters (EC_50_, slope factor): wildtype, 1.1 × 10^−7^ M and 1.3; mutant, 2.2 × 10^−5^ M and 2.3. The resting Po values, estimated from spontaneous activities, are shown on the *left*, at zero concentration. MWC, Monod–Wyman–Changeux; Po, open probability; TRPV1, transient receptor potential vanilloid subfamily, member 1.

Ligand activation of an ion channel has two basic yet functionally linked aspects: how tight the ligand binds and how strong ligand binding promotes activation. These two properties, together with the level of channel activity in the apo state, define the response of an ion channel to ligands (27). In a recent study, we utilized subunit concatenation and irreversible binding of RTX to measure and compare the activation energies from ligand binding to each of the four subunits (24). We found that each subunit contributes the same amount of activation energy, which is 1.70 to 1.86 kcal/mol for RTX. With the binding affinity values obtained in the present study, we are now in the position to make quantitative predictions of the equilibrium properties of TRPV1 activated by capsaicin. With just three parameters (L_0_, K, and f), the model shown in Figure 5*A* satisfactorily predicts concentration-dependent activation of both the wildtype and Y511A mutant channels (Fig. 5*B*). In comparison, the Hill function can describe the high Po data but deviates substantially at the low Po range, as the Hill function yields a straight line in the log-transformed plot, but the experimental data exhibit a clear plateau at low capsaicin concentrations because of unliganded channel openings (Fig. 5*C*). In the middle range of capsaicin concentrations, Hill function yields a more sluggish response, which is more obvious in the wildtype channels.

Successful reproduction of capsaicin activation with the elegantly simple MWC model confirms that TRPV1 is indeed an allosteric protein, as the TRPV1 research community has correctly assumed (28, 29, 30, 31). An allosteric protein exists in a perpetual equilibrium between two functional states—Monod, Wyman, and Changeux used tense (T) and relaxed (R) states to describe hemoglobin (23); for an ion channel, the corresponding states are closed (C) and open (O). Keeping an allosteric protein in a quiescent state under the “resting” physiological condition means that the equilibrium constant for the T←→R balance, L_0_, is sufficiently small. For TRPV1, the L_0_ value under near-physiological conditions (but at room temperature) is in the order of 10^−4^, corresponding to a resting Po of the same order of magnitude that has been experimentally confirmed (12, 24). Binding of vanilloids shifts the C←→O balance toward the open state, by a factor of 17.6 to 23.1 per RTX binding (24) and about 12 per capsaicin binding (Fig. 5*B*). The 1.4 to 1.8 kcal/mol activation energy contributed by each bound ligand explains the high potency of these vanilloid molecules; combination of the contributions from four subunits (yielding 4.8 to 7.2 kcal/mol) is the origin of activation cooperativity. The vanilloid binding steps however occur in a near-independent manner. Being a polymodal nociceptor, it appears TRPV1 may respond to other stimuli (*e.g.*, protons, animal peptide toxins) in a similar manner (32).

Most multisubunit ion channels examined so far exhibit strong cooperativity in their responses to a stimulus (33). Depending on the origin of cooperativity—stimulus sensing *versus* activation gating—a channel’s sensitivity to the stimulus can be quite different (27). Our results from TRPV1 are consistent with the MWC theorem (23) that strong cooperativity can be present even when ligand binding steps are independent: cooperativity of TRPV1 activation originates from the concerted transition, which is promoted equally by each subunit (24). Structures of hemoglobin (34, 35) and TRPV1 (5) reveal that their ligand binding sites are indeed separated. No direct structural interaction is seen in either the apo state or the liganded state. The ligand-binding domains of many ion channels exhibit similar structural features. Cyclic nucleotide–gated channels for example contain four structurally separated intracellular ligand-binding domains (36). Recent studies in the Chanda laboratory found the cyclic nucleotide–binding steps being independent (37) despite functional asymmetry among subunits (38, 39). There are nonetheless ligand-gated ion channels whose ligand-binding domains directly associate. The glutamate receptors, for example, contain four extracellular ligand-binding domains that combine into two structural and functional dimers (40).

Determining L_0_, K, and f for TRPV1 moves one step closer toward understanding the structural mechanism of vanilloid activation. Here, the role of residue Y511 offers a good example. When the first high-resolution TRPV1 structures were solved by the Cheng and Julius laboratories, it was noticed that this aromatic residue, located at the entrance of the vanilloid binding pocket (Fig. 1*A*), switches from a downward position in the apo state to an upward position in the capsaicin- or RTX-bound state (4, 5). Prior mutational studies already identified this residue being highly sensitive in determining capsaicin activation (14, 15). However, despite its close proximity to a bound RTX (9), mutations of Y511 shift capsaicin dependence without substantially reducing the maximal activity (16) or the activation energy exerted by vanilloid binding (12, 24). These observations led us to propose that the upward position of Y511 in the ligand-bound state physically prevents a bound vanilloid molecule from falling out (12). Indeed, mutating Y511 to an amino acid with a smaller side chain such as alanine makes RTX binding reversible (24) and speeds up reversal of capsaicin activation (16); mutating Y511 to a phenylalanine or a tryptophan (with a large nonpolar side chain) does not have as much an effect as changing to a cystine (with a small yet polar side chain) (15, 16), indicating that direct hydrogen bond–type interactions with the bound ligand or nearby residues may not be needed. We showed in this study that Y511A produces a two order of magnitude shift in the capsaicin activation curve by reducing the binding affinity of capsaicin and progressively shifting the concentration dependence for activation (Fig. 5); no major effect on activation energy was detected. It is clear that Y511 functions like a turnstile (revolving gate) to secure vanilloid binding. The knowledge on L_0_, K, and f would open the door to reliably identify the functional role of other channel residues lining the vanilloid binding pocket. Similarly, it is now possible to start investigating how different vanilloid molecules exert distinct functional effects on TRPV1 activity (full or partial agonists or antagonists). For example, whereas RTX is a full agonist, 6′-iRTX is a weak agonist despite their highly similar overall molecular structures (Fig. S2).

## Experimental procedures

### Molecular biology

The wildtype mouse TRPV1 complementary DNA (cDNA) was constructed into the pEYFP-N3 plasmid backbone. The cDNA of enhanced YFP was fused to the C terminus of TRPV1 cDNA to indicate the transfected cells during patch-clamp recordings. The Y512A point mutation was introduced using mutagenesis kit purchased from Agilent Technologies, as previously described (12, 24). All the sequences of these plasmids were confirmed by sequencing. Plasmids of the TRPV1 concatemers (YYYY, YYYA, YYAA, YAAA, and AAAA) were generated by Dr Avi Priel as previously described (25). Briefly, rat TRPV1 wildtype and Y511A (equivalent to Y512A in the mouse TRPV1 channel) cDNA segments were linked with sequences encoding flexible peptide linkers to build the tandem tetrameric channel plasmids. It was observed that the activation profile of channels with four concatenated wildtype subunits closely mimicked that of channels made with monomeric wildtype subunits (25).

### Cell culture

TSA201 (human embryonic kidney 293T) cells were purchased from American Type Culture Collection and used for mouse and rat TRPV1 expression and patch-clamp recordings. Cells were cultured in Dulbecco's modified Eagle's high glucose medium (Gibco) with 10% (v/v) fetal bovine serum (GenClone) at 37°C with 5% CO_2_. Cells grown to 30% to 50% confluence on 25 mm glass coverslips in 30 mm dishes (Fisher Scientific) were used for transient transfection using Lipofectamine 2000 (Invitrogen) according to the manufacturer’s instructions. Patch-clamp experiments were conducted 18 to 24 h after transfection. For macroscopic recordings, 1 μg plasmids were used for each 30 mm dish; for single-channel recordings, 0.1 μg plasmids were used for each 30 mm dish. Because the TRPV1 concatemer plasmids did not contain the fluorescent tag, 0.2 μg YFP plasmids were cotransfected to identify transfected cells for patch-clamp experiments.

### Chemical solutions

Symmetrical bath and pipette solutions were used for patch-clamp recordings, which contained (in millimolar) 140 NaCl, 2 EDTA, and 15 Hepes (pH 7.4). Capsaicin (Sigma–Aldrich) was dissolved in dimethyl sulfoxide to make the 1 mM stock solution and diluted to working concentrations (0.01 to 100 μM) using bath solution. Since it is known that high concentrations of capsaicin produce nonspecific effects on membrane and embedded proteins (41), higher concentrations of capsaicin were not attempted in this study. 2-APB (purchased from Sigma–Aldrich) was dissolved in dimethyl sulfoxide to make the 1 M stock solution and diluted to the 3 mM working concentration using the bath solution. 6-iRTX (purchased from Sigma–Aldrich) and RTX (purchased from Alomone Labs) were dissolved in ethanol to make the 1 mM stock solution and diluted to working concentrations (10 to 200 nM) using the bath solution. A 100 mM BaCl_2_ solution with 15 mM Hepes was used to block TRPV1 current from the intracellular side.

### Electrophysiology

Patch-clamp pipettes were pulled from borosilicate glass tubes (Sutter Instrument) using a Sutter Instrument P-97 micropipette puller and fire-polished to create resistances of 2 to 6 MΩ for macroscopic recordings or 8 to 15 MΩ for single-channel recordings. An EPC 10 USB patch-clamp amplifier controlled by PatchMaster software (HEKA Elektronik) was used. The current signal was sampled at 10 kHz and filtered at 2.25 kHz. The holding potential was set at 0 mV, followed by a 500 ms step to +80 mV and then a 500 ms step to −80 mV. The durations of the +80 mV and −80 mV steps were adjusted between 300 ms and 2 s as per experimental requirements. For continuous single-channel recordings, the voltage was held at +80 mV throughout the entire recording. Perfusion was gravity driven, and solution switching was executed using a Rapid Solution Changer (RSC-200; Biological Science Instruments).

### Data analysis

Patch-clamp data were exported from the PatchMaster software and analyzed using Igor Pro 8 (WaveMetrics). Statistical analyses were performed using GraphPad Prism 8 (GraphPad Software, Inc). The Student's *t* test was employed when comparing two groups. Paired *t* tests were used for comparisons within the same recordings. For comparison among multiple groups, one-way ANOVA was utilized.

Macroscopic current amplitude was calculated by measuring the difference between ligand-activated current and the baseline current level before any ligand perfusion. A digital filter at 0.4 kHz was used for analyzing single-channel amplitude and Po. Single-channel recordings were analyzed using all-point histograms and fitted to a double-Gaussian function; single-channel Po was measured by calculating the portion of open events over the total time recorded. Only true single-channel recordings or two-channel recordings were used. Po of two-channel recordings was calculated using the equation $Po=\frac{t_{1}+2\times t_{2}}{2\times T}$, where $t_{1}$ is the total time of one-channel opening evens observed, $t_{2}$ is the total time of two-channel opening evens observed, and *T* is the total recording time. Spontaneous Po was measured from single-channel recordings without perfusing any ligands, and a saturating concentration of 2-APB (3 mM) or capsaicin (10–100 μM) was used to determine the number of channels in the patch.

For the YYYA concatemer channels preloaded with three 6′-iRTX molecules in the Y subunits, the single-channel Po data were fitted with the MWC model shown in Figure 1*B*, using the equation $Po=\frac{L_{3}\;+\;fK\left[c\right]L_{3}}{1\;+\;K\left[c\right]\;+\;L_{3}\;+\;fK\left[c\right]L_{3}}$, in which $L_{3}$ is the equilibrium constant before capsaicin application, f is the cooperative factor describing the fold increase of the equilibrium constant by capsaicin, K is the capsaicin association constant for the A subunit, and $\left[c\right]$ is the concentration of capsaicin.

For the YYAA concatemer channels preloaded with two RTX molecules in the Y subunits, the single-channel Po data were fitted with the MWC model shown in Figure 2*A*, using the equation $Po=\frac{L_{2}+2fK\left[c\right]L_{2}+f^{2}K^{2}{\left[c\right]}^{2}L_{2}}{1+L_{2}+2K\left[c\right]+K^{2}{\left[c\right]}^{2}+2fK\left[c\right]L_{2}+f^{2}K^{2}{\left[c\right]}^{2}L_{2}}$, where $L_{2}$ is the equilibrium constant before capsaicin application, f, K, and $\left[c\right]$ are the same as defined previously. Alternatively, two separate associate constants K1 and K2 were assumed for the first and seconding binding steps. The Po data were fitted with the MWC model shown in Figure 3*A*, using the equation $Po=\frac{L_{2}+fK^{\prime }\left[c\right]L_{2}+f^{2}a{K^{\prime }}^{2}{\left[c\right]}^{2}L_{2}}{1+L_{2}+K^{\prime }\left[c\right]+a{K^{\prime }}^{2}{\left[c\right]}^{2}+fK^{\prime }\left[c\right]L_{2}+f^{2}a{K^{\prime }}^{2}{\left[c\right]}^{2}L_{2}}$, where the factor a would be 0.25 when the two binding steps are independent. Global fittings were conducted in Igor Pro using the equations described previously, with each fitting parameter(s) being either specific to one dataset or shared among datasets.

For YYYY, YYYA, YYAA, YAAA, and AAAA concatemer channels activated by capsaicin without pretreatment with RTX or 6′-iRTX, the single-channel Po data were fitted with the MWC model shown in Figure 4*A*. Given that each capsaicin binding step is independent, capsaicin binding to a wildtype subunit was assumed to have the same association constant *K*_Y_, whereas binding to a mutant subunit was assumed to have the same binding affinity *K*_A_.. A global fitting routine was conducted in Igor Pro, using the following equation:

$$Po=\frac{\left(1+\sum_{i=1}^{4}fK_{i}\left[c\right]+\sum_{1\leq i<j\leq 4}f^{2}K_{i}K_{j}{\left[c\right]}^{2}+\sum_{1\leq i<j<l\leq 4}f^{3}K_{i}K_{j}K_{l}{\left[c\right]}^{3}+f^{4}K_{1}K_{2}K_{3}K_{4}{\left[c\right]}^{4}\right)L}{\left(1+\sum_{i=1}^{4}fK_{i}\left[c\right]+\sum_{1\leq i<j\leq 4}f^{2}K_{i}K_{j}{\left[c\right]}^{2}+\sum_{1\leq i<j<l\leq 4}f^{3}K_{i}K_{j}K_{l}{\left[c\right]}^{3}+f^{4}K_{1}K_{2}K_{3}K_{4}{\left[c\right]}^{4}\right)L+1+\sum_{i=1}^{4}K_{i}\left[c\right]+\sum_{1\leq i<j\leq 4}K_{i}K_{j}{\left[c\right]}^{2}+\sum_{1\leq i<j<l\leq 4}K_{i}K_{j}K_{l}{\left[c\right]}^{3}+K_{1}K_{2}K_{3}K_{4}{\left[c\right]}^{4}}$$

in which L is the equilibrium constant without capsaicin, $K_{1}\text{,}$ $K_{2},K_{3},K_{4}$ are the association constant for the first, second, third, and fourth subunit, respectively, f and $\left[c\right]$ are as defined previously. For the YYYY concatemer, $K_{1}=K_{2}=K_{3}=K_{4}=K_{Y}\text{;}$ for the YYAA concatemer, $K_{1}=K_{2}=K_{Y},K_{3}=K_{4}=K_{A}$; and so on.

## Data and materials availability

All the data supporting this study are available in the main text or the supporting information.

## Supporting information

This article contains supporting information.

## Conflict of interest

The authors declare that they have no conflicts of interest with the contents of this article.

## Supporting information

CAP k figures 6

CAP k figures 7

CAP k figures 8

CAP k figures 9

CAP k figures 10

CAP k figures 11

CAP k figures 12

CAP k figures 13

CAP k figures 14

## References

[1] Caterina M.J., Schumacher M.A., Tominaga M., Rosen T.A., Levine J.D., Julius D. (1997). The capsaicin receptor: a heat-activated ion channel in the pain pathway. Nature.

[2] Tominaga M., Caterina M.J., Malmberg A.B., Rosen T.A., Gilbert H., Skinner K. (1998). The cloned capsaicin receptor integrates multiple pain-producing stimuli. Neuron.

[3] Yang F., Zheng J. (2017). Understand spiciness: mechanism of TRPV1 channel activation by capsaicin. Protein Cell.

[4] Cao E., Liao M., Cheng Y., Julius D. (2013). TRPV1 structures in distinct conformations reveal activation mechanisms. Nature.

[5] Liao M., Cao E., Julius D., Cheng Y. (2013). Structure of the TRPV1 ion channel determined by electron cryo-microscopy. Nature.

[6] Zhang K., Julius D., Cheng Y. (2021). Structural snapshots of TRPV1 reveal mechanism of polymodal functionality. Cell.

[7] Kwon D.H., Zhang F., Fedor J.G., Suo Y., Lee S.-Y. (2022). Vanilloid-dependent TRPV1 opening trajectory from cryoEM ensemble analysis. Nat. Commun..

[8] Neuberger A., Oda M., Nikolaev Y.A., Nadezhdin K.D., Gracheva E.O., Bagriantsev S.N., Sobolevsky A.I. (2023). Human TRPV1 structure and inhibition by the analgesic SB-366791. Nat. Commun..

[9] Gao Y., Cao E., Julius D., Cheng Y. (2016). TRPV1 structures in nanodiscs reveal mechanisms of ligand and lipid action. Nature.

[10] Hui K., Liu B., Qin F. (2003). Capsaicin activation of the pain receptor, VR1: multiple open states from both partial and full binding. Biophys. J..

[11] Yang F., Xiao X., Lee B.H., Vu S., Yang W., Yarov-Yarovoy V., Zheng J. (2018). The conformational wave in capsaicin activation of transient receptor potential vanilloid 1 ion channel. Nat. Commun..

[12] Yang F., Xiao X., Cheng W., Yang W., Yu P., Song Z. (2015). Structural mechanism underlying capsaicin binding and activation of the TRPV1 ion channel. Nat. Chem. Biol..

[13] Salazar H., Jara-Oseguera A., Hernández-García E., Llorente I., Arias-Olguín I.I., Soriano-García M. (2009). Structural determinants of gating in the TRPV1 channel. Nat. Struct. Mol. Biol..

[14] Gavva N.R., Klionsky L., Qu Y., Shi L., Tamir R., Edenson S. (2004). Molecular determinants of vanilloid sensitivity in TRPV1. J. Biol. Chem..

[15] Jordt S.-E., Julius D. (2002). Molecular basis for species-specific sensitivity to “hot” chili peppers. Cell.

[16] Hazan A., Basu A., Zalcman N., Matzner H., Priel A., Priel A. (2016). Tyrosine residue in the TRPV1 vanilloid binding pocket regulates deactivation kinetics. J. Biol. Chem..

[17] Yin Y., Dong Y., Vu S., Yang F., Yarov-Yarovoy V., Tian Y., Zheng J. (2019). Structural mechanisms underlying activation of TRPV1 channels by pungent compounds in gingers. Br. J. Pharmacol..

[18] Vu S., Singh V., Wulff H., Yarov-Yarovoy V., Zheng J. (2020). New capsaicin analogs as molecular rulers to define the permissive conformation of the mouse TRPV1 ligand-binding pocket. Elife.

[19] Dong Y., Yin Y., Vu S., Yang F., Yarov-Yarovoy V., Tian Y., Zheng J. (2019). A distinct structural mechanism underlies TRPV1 activation by piperine. Biochem. Biophys. Res. Commun..

[20] Elokely K., Velisetty P., Delemotte L., Palovcak E., Klein M.L., Rohacs T., Carnevale V. (2016). Understanding TRPV1 activation by ligands: insights from the binding modes of capsaicin and resiniferatoxin. Proc. Natl. Acad. Sci. U. S. A..

[21] Darré L., Domene C. (2015). Binding of capsaicin to the TRPV1 ion channel. Mol. Pharmaceutics.

[22] Colquhoun D. (1998). Binding, gating, affinity and efficacy: the interpretation of structure-activity relationships for agonists and of the effects of mutating receptors. Br. J. Pharmacol..

[23] Monod J., Wyman J., Changeux J.P. (1965). On the nature of allosteric transitions: a plausible model. J. Mol. Biol..

[24] Li S., Nguyen P.T., Vu S., Yarov-Yarovoy V., Zheng J. (2023). Opening of capsaicin receptor TRPV1 is stabilized equally by its four subunits. J. Biol. Chem..

[25] Hazan A., Kumar R., Matzner H., Priel A. (2015). The pain receptor TRPV1 displays agonist-dependent activation stoichiometry. Sci. Rep..

[26] McDonnell M.E., Zhang S.-P., Dubin A.E., Dax S.L. (2002). Synthesis and *in vitro* evaluation of a novel iodinated resiniferatoxin derivative that is an agonist at the human vanilloid VR1 receptor. Bioorg. Med. Chem. Lett..

[27] Zagotta W.N. (2023). Textbook of Ion Channels.

[28] Latorre R., Brauchi S., Orta G., Zaelzer C., Vargas G. (2007). ThermoTRP channels as modular proteins with allosteric gating. Cell Calcium.

[29] Matta J.A., Ahern G.P. (2007). Voltage is a partial activator of rat thermosensitive TRP channels. J. Physiol..

[30] Jara-Oseguera A., Islas L.D. (2013). The role of allosteric coupling on thermal activation of thermo-TRP channels. Biophys. J..

[31] Cao X., Ma L., Yang F., Wang K., Zheng J. (2014). Divalent cations potentiate TRPV1 channel by lowering the heat activation threshold. J. Gen. Physiol..

[32] Zheng J. (2013). Molecular mechanism of TRP channels. Compr. Physiol..

[33] Zheng J., Trudeau M.C. (2023). Textbook of Ion Channels: Three Volume Set.

[34] Bolton W., Perutz M. (1970). Three dimensional Fourier synthesis of horse deoxyhaemoglobin at 2.8 Å resolution. Nature.

[35] Shaanan B. (1983). Structure of human oxyhaemoglobin at 2· 1resolution. J. Mol. Biol..

[36] M. D. Varnum, D. Gucan, "Cyclic nucleotide-gated channels" in Textbook of Ion Channels Volume II. (CRC Press), pp. 163-180.

[37] White D.S., Chowdhury S., Idikuda V., Zhang R., Retterer S.T., Goldsmith R.H., Chanda B. (2021). cAMP binding to closed pacemaker ion channels is non-cooperative. Nature.

[38] Liu D.T., Tibbs G.R., Paoletti P., Siegelbaum S.A. (1998). Constraining ligand-binding site stoichiometry suggests that a cyclic nucleotide–gated channel is composed of two functional dimers. Neuron.

[39] Schirmeyer J., Hummert S., Eick T., Schulz E., Schwabe T., Ehrlich G. (2021). Thermodynamic profile of mutual subunit control in a heteromeric receptor. Proc. Natl. Acad. Sci. U. S. A..

[40] Plested A. (2023). Ionotropic glutamate receptors. Textbook Ion Channels Volume Properties, Funct. Pharmacol. Superfamilies.

[41] Ingólfsson H.I., Thakur P., Herold K.F., Hobart E.A., Ramsey N.B., Periole X. (2014). Phytochemicals perturb membranes and promiscuously alter protein function. ACS Chem. Biol..

