# Supplementary figures and images for: The capsaicin binding affinity of wildtype and mutant TRPV1 ion channels
Source: J Biol Chem. 2023 Sep 20;299(11):105268. doi: 10.1016/j.jbc.2023.105268 (PMC10616419; doi:10.1016/j.jbc.2023.105268)

SF1. MWC allosteric model

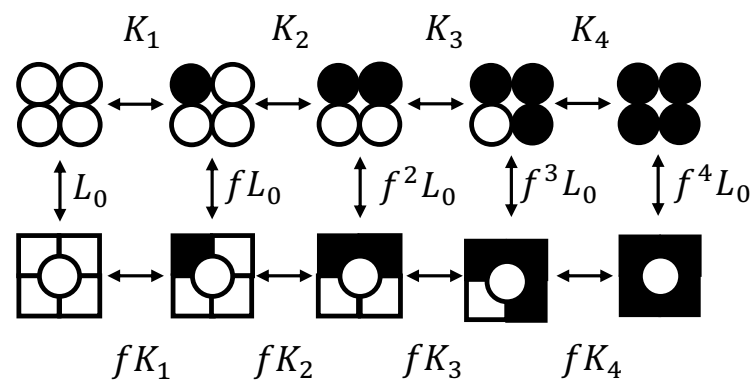

Supplement: CAP k figures 6 [file mmc1.pdf]

**SF2.** Structure comparison between 6'-iRTX and RTX

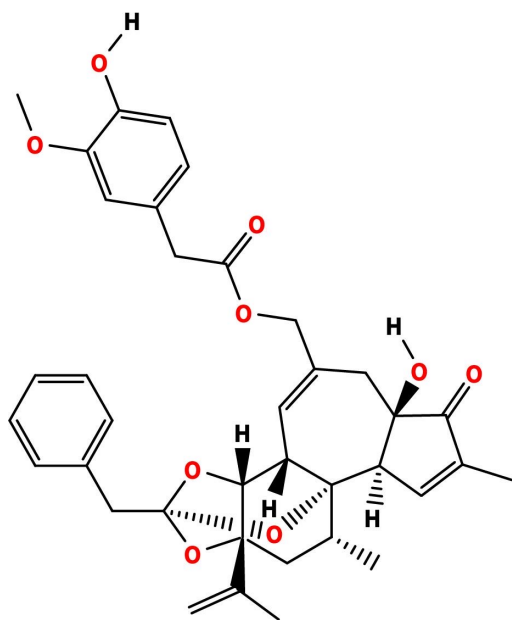

RTX

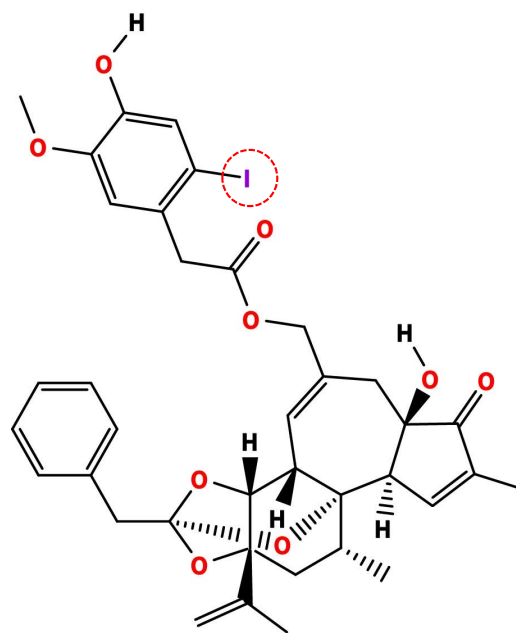

6'-iRTX

Supplement: CAP k figures 7 [file mmc2.pdf]

**SF3.** 6'-iRTX irreversibly bound to WT mouse TRPV1 in single channel recording

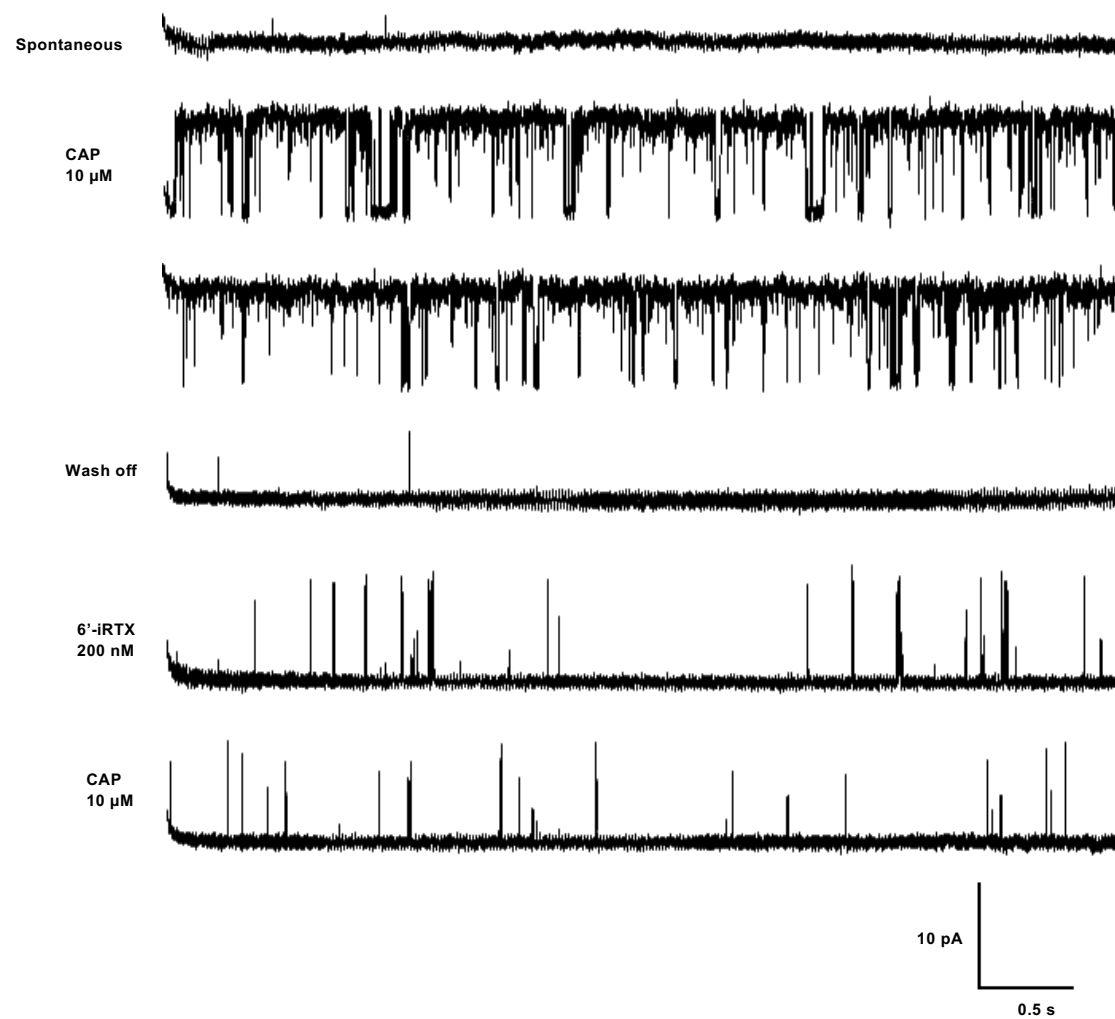

Supplement: CAP k figures 8 [file mmc3.pdf]

**SF4.** 6'-iRTX reversibly bound to Y512A mouse TRPV1 in single channel recording

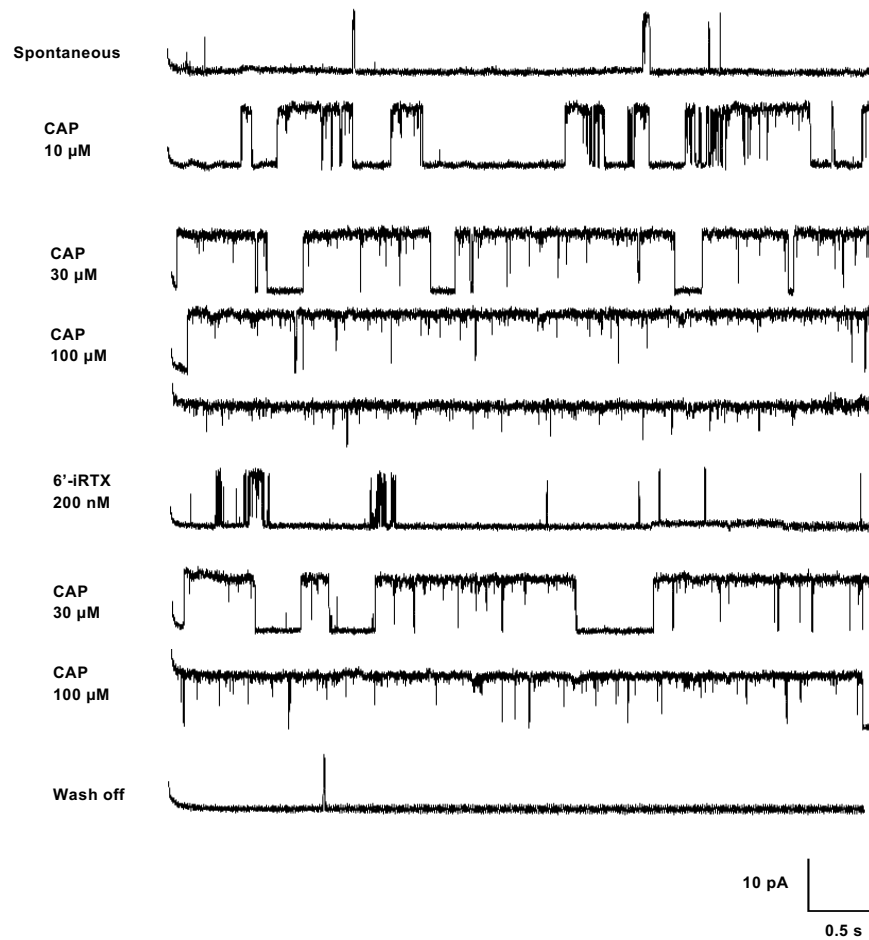

Supplement: CAP k figures 9 [file mmc4.pdf]

**SF8a.** Capsaicin response in rat TRPV1 concatemers in single channel recording

YYYY CAP

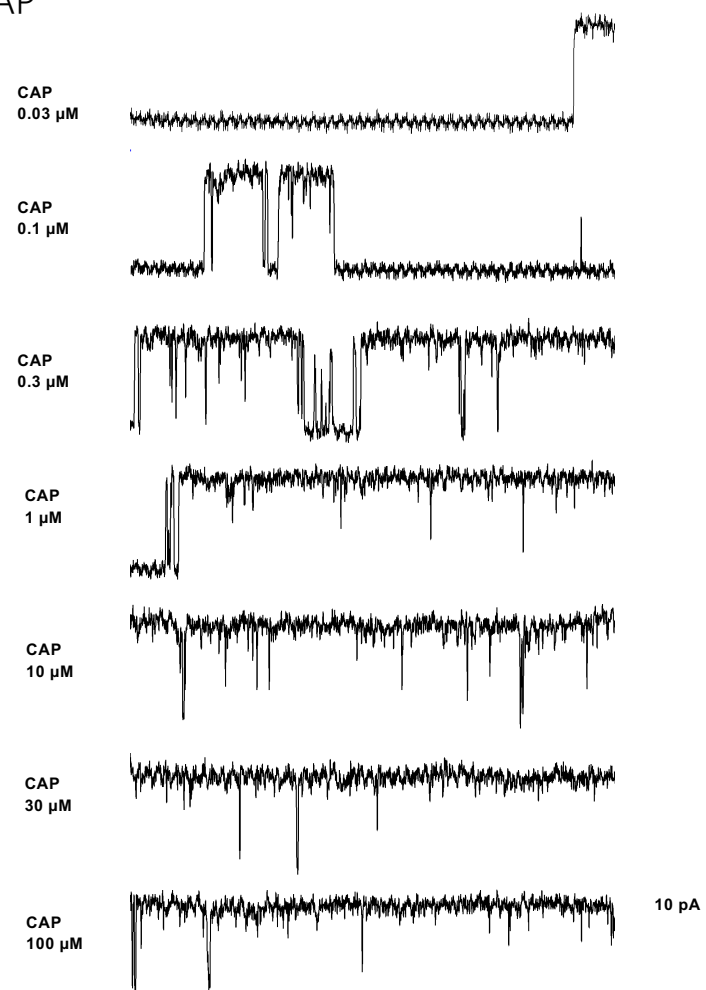

YYYA CAP

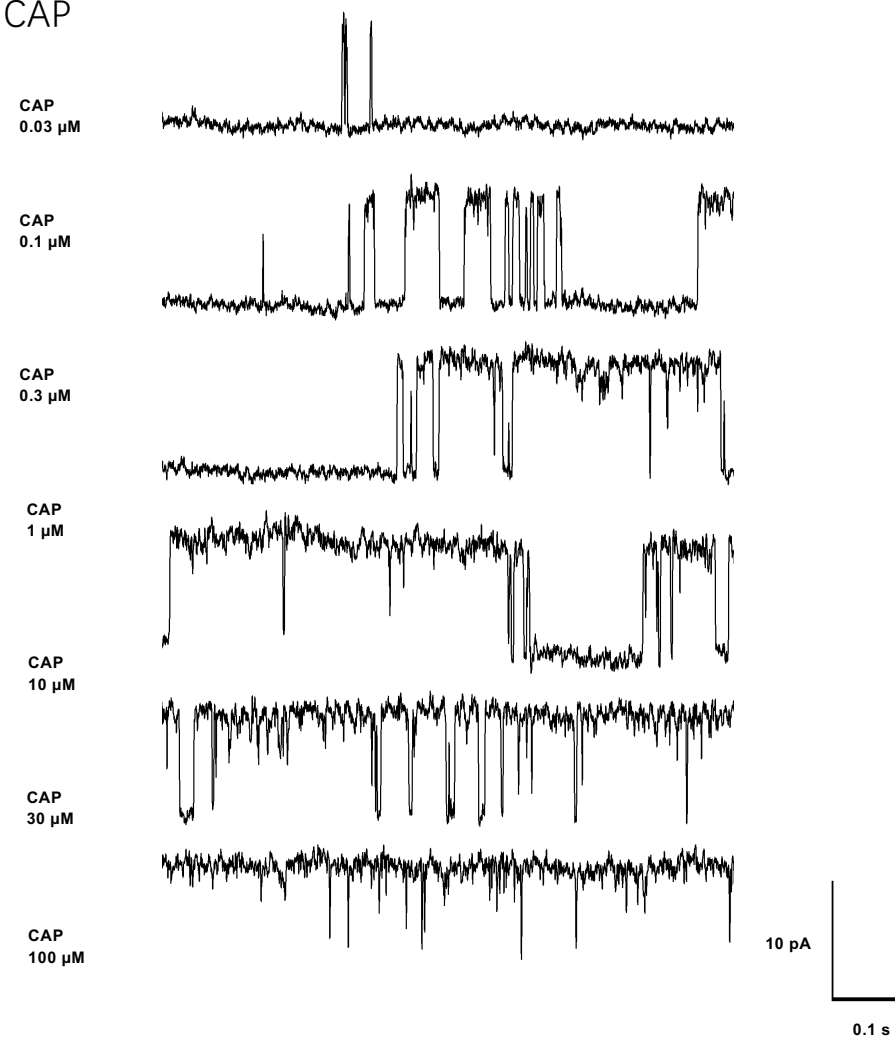

Supplement: CAP k figures 13 [file mmc8.pdf]
