# Supplementary material for: The capsaicin binding affinity of wildtype and mutant TRPV1 ion channels
Source: J Biol Chem. 2023 Sep 20;299(11):105268. doi: 10.1016/j.jbc.2023.105268 (PMC10616419; doi:10.1016/j.jbc.2023.105268)

SF6. Capsaicin response in YYAA rat TRPV1 with preloaded 2 RTX molecules in single channel recording

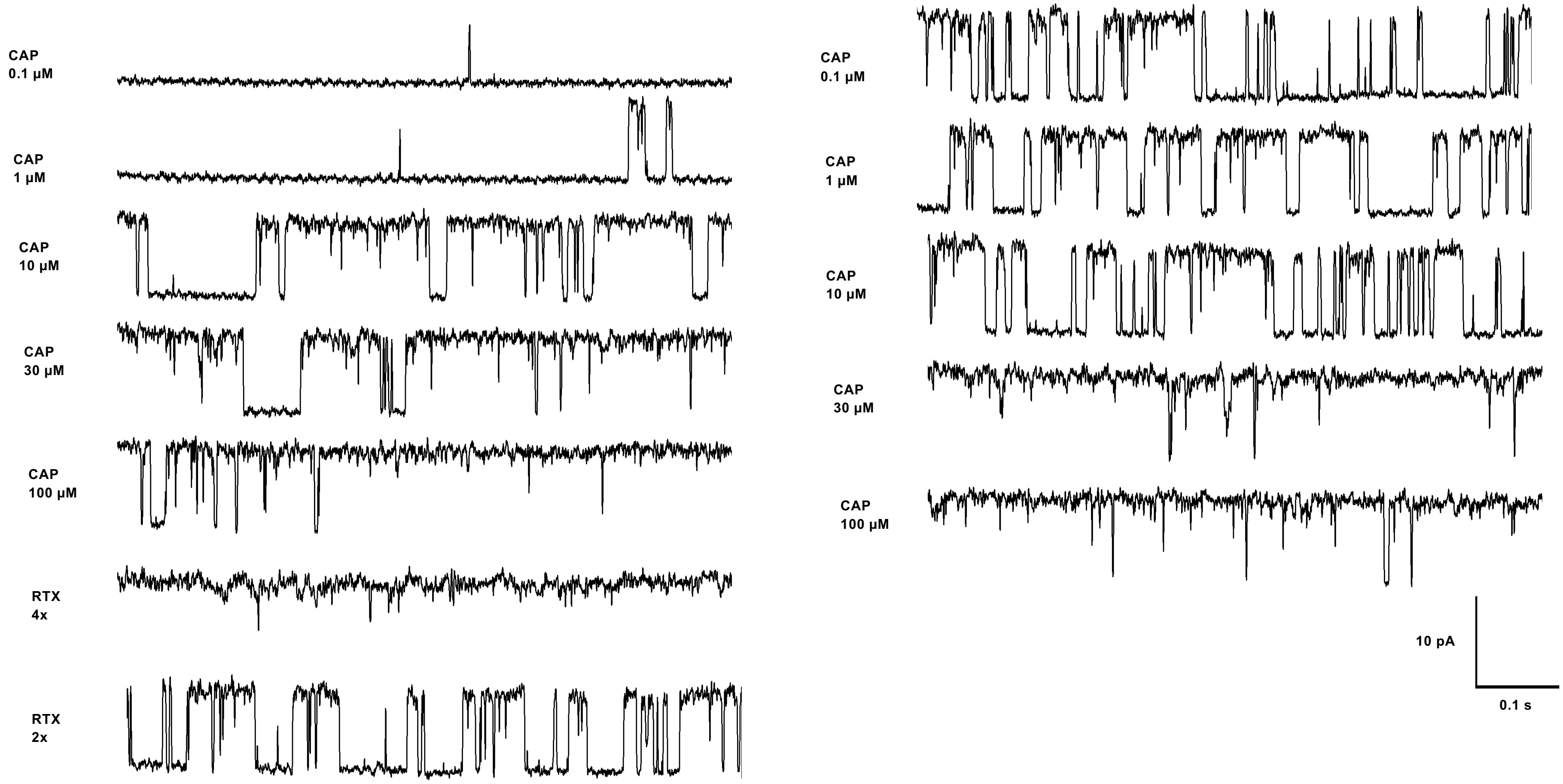

Supplement: CAP k figures 11 [file mmc6.pdf]
